# The Year-Book of Treatment for 1891

**Published:** 1891-05

**Authors:** 


THE YEAR-BOOK OF TREATMENT FOR 1891. Lea Brothers & Co. Philadelphia.

This is one of the most popular books of the series system that is published. The publishers have increased considerably the size of the book for this year. The book is so well divided in parts that it enables the busy practitioner to utilize his leisure moments so as to keep fully abreast with the most advanced literature of the day upon all subjects pertaining to medicine. The book is not intended as a dictionary or text-book of medicine. We most heartily commend it to the wide-awake, progressive doctor who wishes to know what is being written each year by the best men of the profession. W. A. C.

